# The interplay of family history of depression and early trauma: associations with lifetime and current depression in the German national cohort (NAKO)

**DOI:** 10.3389/fepid.2023.1099235

**Published:** 2023-05-23

**Authors:** Fabian Streit, Maja P. Völker, Johanna Klinger-König, Lea Zillich, Josef Frank, Iris Reinhard, Jerome C. Foo, Stephanie H. Witt, Lea Sirignano, Heiko Becher, Nadia Obi, Oliver Riedel, Stefanie Do, Stefanie Castell, Max J. Hassenstein, André Karch, Andreas Stang, Börge Schmidt, Tamara Schikowski, Anna Stahl-Pehe, Hermann Brenner, Laura Perna, Karin Halina Greiser, Rudolf Kaaks, Karin B. Michels, Claus-Werner Franzke, Annette Peters, Beate Fischer, Julian Konzok, Rafael Mikolajczyk, Amand Führer, Thomas Keil, Julia Fricke, Stefan N. Willich, Tobias Pischon, Henry Völzke, Claudia Meinke-Franze, Markus Loeffler, Kerstin Wirkner, Klaus Berger, Hans J. Grabe, Marcella Rietschel

**Affiliations:** ^1^ Department of Genetic Epidemiology in Psychiatry, Central Institute of Mental Health, Medical Faculty Mannheim, Heidelberg University, Mannheim, Germany; ^2^ Department of Psychiatry and Psychotherapy, University Medicine Greifswald, Greifswald, Germany; ^3^ Department of Biostatistics, Central Institute of Mental Health, Medical Faculty Mannheim, Heidelberg University, Mannheim, Germany; ^4^ Institute of Global Health, University Hospital Heidelberg, Heidelberg, Germany; ^5^ Institute of Medical Biometry and Epidemiology, University Medical Centre Hamburg-Eppendorf, Hamburg, Germany; ^6^ Leibniz-Institut für Präventionsforschung und Epidemiologie – BIPS, Bremen, Deutschland; ^7^ Department for Epidemiology, Helmholtz Centre for Infection Research (HZI), Braunschweig, Germany; ^8^ PhD Programme “Epidemiology”, Braunschweig-Hannover, Germany; ^9^ Institute of Epidemiology and Social Medicine, University of Muenster, Muenster, Germany; ^10^ Institute for Medical Informatics, Biometry and Epidemiology, University of Duisburg-Essen, Essen, Germany; ^11^ IUF—Leibniz Institute for Environmental Medicine, Düsseldorf, Germany; ^12^ Institute for Biometrics and Epidemiology, German Diabetes Center, Leibniz Center for Diabetes Research, University of Düsseldorf, Düsseldorf, Germany; ^13^ Network Ageing Research (NAR), Heidelberg University, Heidelberg, Germany; ^14^ Division of Clinical Epidemiology & Ageing Research, German Cancer Research Centre (DKFZ), Heidelberg, Germany; ^15^ Department of Translational Research in Psychiatry, Max Planck Institute of Psychiatry, Munich, Germany; ^16^ German Cancer Research Centre (DKFZ) Heidelberg, Div. of Cancer Epidemiology, Heidelberg, Germany; ^17^ Institute for Prevention and Cancer Epidemiology, Faculty of Medicine and Medical Center, University of Freiburg, Freiburg, Germany; ^18^ Institute of Epidemiology, Helmholtz Zentrum Munchen, German Research Centre for Environmental Health, Neuherberg, Germany; ^19^ Chair of Epidemiology, Institute for Medical Information Processing, Biometry and Epidemiology, Medical Faculty, Ludwig-Maximilians-Universität München, Munich, Germany; ^20^ Department of Epidemiology and Preventive Medicine, University of Regensburg, Regensburg, Germany; ^21^ Institute for Medical Epidemiology, Biometrics and Informatics (IMEBI), Interdisciplinary Centre for Health Sciences, Medical School of the Martin Luther University Halle-Wittenberg, Halle, Germany; ^22^ German Center for Mental Health, Site Jena-Magdeburg-Halle, Jena, Germany; ^23^ Institute of Social Medicine, Epidemiology and Health Economics, Charité - Universitätsmedizin Berlin, Berlin, Germany; ^24^ Institute for Clinical Epidemiology and Biometry, University of Wuerzburg, Wuerzburg, Germany; ^25^ State Institute of Health, Bavarian Health and Food Safety Authority, Bad Kissingen, Germany; ^26^ Max-Delbrueck-Centre for Molecular Medicine in the Helmholtz Association (MDC), Molecular Epidemiology Research Group, Berlin, Germany; ^27^ Charité – Universitätsmedizin Berlin, Corporate Member of Freie Universität Berlin and Humboldt-Universität zu Berlin, Berlin, Germany; ^28^ Max-Delbrueck-Centre for Molecular Medicine in the Helmholtz Association (MDC), Biobank Technology Platform, Berlin, Germany; ^29^ Institute for Community Medicine, University Medicine Greifswald, Greifswald, Germany; ^30^ Institute for Community Medicine, Section Epidemiology of Health Care and Community Health, University Medicine Greifswald (UMG), Greifswald, Germany; ^31^ German Centre for Neurodegenerative Diseases (DZNE) Site Rostock/Greifswald, Greifswald, Germany; ^32^ Institute for Medical Informatics, Statistics and Epidemiology (IMISE), University of Leipzig, Leipzig, Germany; ^33^ Leipzig Research Center for Civilization Diseases (LIFE), University of Leipzig, Leipzig, Germany; ^34^ Institute of Epidemiology & Social Medicine, University of Muenster, Muenster, Germany

**Keywords:** depression, childhood trauma, family history, genetics, abuse, neglect, maltreatment

## Abstract

**Introduction:**

Family history of depression and childhood maltreatment are established risk factors for depression. However, how these factors are interrelated and jointly influence depression risk is not well understood. The present study investigated (i) if childhood maltreatment is associated with a family history of depression (ii) if family history and childhood maltreatment are associated with increased lifetime and current depression, and whether both factors interact beyond their main effects, and (iii) if family history affects lifetime and current depression via childhood maltreatment.

**Methods:**

Analyses were based on a subgroup of the first 100,000 participants of the German National Cohort (NAKO), with complete information (58,703 participants, mean age = 51.2 years, 53% female). Parental family history of depression was assessed via self-report, childhood maltreatment with the Childhood Trauma Screener (CTS), lifetime depression with self-reported physician's diagnosis and the Mini-International Neuropsychiatric Interview (MINI), and current depressive symptoms with the depression scale of the Patient Health Questionnaire (PHQ-9). Generalized linear models were used to test main and interaction effects. Mediation was tested using causal mediation analyses.

**Results:**

Higher frequencies of the childhood maltreatment measures were found in subjects reporting a positive family history of depression. Family history and childhood maltreatment were independently associated with increased depression. No statistical interactions of family history and childhood maltreatment were found for the lifetime depression measures. For current depressive symptoms (PHQ-9 sum score), an interaction was found, with stronger associations of childhood maltreatment and depression in subjects with a positive family history. Childhood maltreatment was estimated to mediate 7%–12% of the effect of family history on depression, with higher mediated proportions in subjects whose parents had a depression onset below 40 years. Abuse showed stronger associations with family history and depression, and higher mediated proportions of family history effects on depression than neglect.

**Discussion:**

The present study confirms the association of childhood maltreatment and family history with depression in a large population-based cohort. While analyses provide little evidence for the joint effects of both risk factors on depression beyond their individual effects, results are consistent with family history affecting depression via childhood maltreatment to a small extent.

## 1. Introduction

Depression is one of the most common mental illnesses worldwide (1), and approximately one in five people will develop depression in their lifetime (2) with women being nearly twice as likely to be affected (3). Compared to other mental disorders, depressive disorders impose the highest burden of disease on society with the largest proportion of disability-adjusted life years (4). Affected individuals report impaired daily functioning and reduced quality of life (5). Both genetic and environmental conditions play an important role in the etiology of depression. In the present study, we aim to assess the interplay of these factors, by examining the joint effects of a history of parental depression and childhood maltreatment on current and lifetime depression.

### 1.1. Family history of depression

Depression runs in families and formal genetic studies have shown a substantial heritability of approximately 40% (6). A positive family history of depression (i.e., one or more family members have been affected with depression during their lifetimes) considerably increases the risk for depression, especially if first-degree relatives are affected (7–9). Additionally, a younger age at onset and a higher number of affected family members have been found to further increase the risk for depression (7–10). Since family history of depression is currently more informative of depression risk than the results of genetic testing, family history has been widely used in genetic counseling settings (11–13). It should be noted that while family history of depression can be used as a marker for genetic risk in clinical practice and research, it also comprises exposure related to environmental and lifestyle factors shared within the family (14).

### 1.2. Early maltreatment

Stressful and traumatic life experiences have been identified as important risk factors for depression with childhood being a particularly vulnerable phase (3, 15–17). It has been estimated that more than half of global cases of depression may be attributable to the experience of childhood maltreatment (18). Individuals with a history of childhood maltreatment are at an increased risk of developing not only depression but also other negative health outcomes (e.g., high blood pressure, drug abuse) compared to those without childhood maltreatment (19–21). In patients with depression, childhood maltreatment has been linked to greater comorbidities and lower treatment response (22), the experience of recurrent episodes (23), and a chronic course (24). The impact of childhood maltreatment on depression might vary according to the exact type of childhood maltreatment, e.g., emotional abuse and emotional neglect were found to have stronger associations with depression diagnosis and symptom severity compared to sexual abuse, physical abuse, and physical neglect (25).

### 1.3. The interplay of family history of depression and early maltreatment

A family history of depression and childhood maltreatment cannot be considered independent risk factors, especially if family history assessment focuses on the parents. Usually, parents as the main caregivers for their children are key in shaping the environments of their children during childhood and adolescence (26, 27). Studies show that childhood maltreatment is more frequent in subjects with a positive family history of mental disorders such as mood disorders, psychotic disorders, and posttraumatic stress disorder [e.g., (17, 28–30)].

Furthermore, individuals may respond very differently to the experience of childhood maltreatment (31). As such, individuals with a predisposition for mental disorders, e.g., with a positive family history of depression, might respond more strongly to adverse events (32). While it has been demonstrated in joint models that a positive family history of depression and childhood maltreatment each have effects on the risk of depression (16), it is unclear to what extent an interaction between these two conditions contributes to the disorder risk beyond their individual effects.

Additionally, it has been postulated that childhood maltreatment mediates the association between family history of depression and the offspring's depression, i.e., a positive family history of depression might influence the risk for depression via childhood maltreatment. However, little research has been carried out to test this. One study by Jansen and colleagues investigated the mediation effects of childhood maltreatment in patients with major depressive disorder or bipolar disorder, finding that up to 35% of the effects of family history of affective disorders on the offspring's affective disorder were mediated by childhood maltreatment (17).

In summary, while both a positive family history of depression and childhood maltreatment represent well-established risk factors for depression, it is not sufficiently understood how family history of depression and childhood maltreatment act together on depression risk. For robust estimations of such effects, large samples are required (33). Therefore, large-scale cohorts represent a valuable resource for such investigations. In the German National Cohort (NAKO Gesundheitsstudie, NAKO), a prospective population-based study aiming to investigate the causes and preclinical stages of common chronic illnesses (34, 35), associations of family history of depression and childhood maltreatment with depression have been demonstrated separately: parental history of depression was associated with higher frequencies of lifetime depression and stronger current depressive symptoms, with the association of family history of depression with depression being most pronounced in individuals with both parents affected (36). Childhood maltreatment and its categories of abuse and neglect were associated with stronger current depressive symptoms (37). The specific maltreatment items (physical abuse, emotional abuse, sexual abuse, physical neglect, emotional neglect) showed varying correlations with each other (r range = 0.07–0.39), hinting at distinct properties of childhood maltreatment types that might impact mental health outcomes differently. However, associations with depression were only assessed on a categorical level for abuse and neglect.

### 1.4. Aims

The present study investigated the interplay between family history of depression and childhood maltreatment in depression in the NAKO cohort. We aimed to test (i) if a higher frequency of childhood maltreatment is reported by participants with a family history of depression (ii) if family history of depression and childhood maltreatment are associated with increased lifetime and current depression, and whether the two factors interact in their association with depression beyond their main effects, and (iii) if family history of depression affects lifetime and current depression via childhood maltreatment. We examined those associations for different childhood maltreatment types as well as characteristics of family history of depression such as the number of affected parents and earliest parental age at onset.

## 2. Methods

### 2.1. Sample

For the present analyses, data from the NAKO cohort was used. Baseline assessment was performed in 18 study centers across Germany between 2014 and 2019 (34, 35, 38). The study was approved by the ethical committees of the study centers and written consent was obtained from the participants. Subjects were eligible for participation if their primary residence was located within the respective study region. They were excluded from the study if they were incapable of giving informed consent, understanding the study information, responding to the questions (e.g., due to language barriers), or participating in the majority of examinations (34, 35, 38, 39).

At baseline, 205,415 participants between the ages of 20–69 were recruited, of which 362 (0.18%) subjects revoked their consent (34, 35). The final sample included 205,053 participants (35). The assessments included physical examinations, a guided face-to-face interview with a trained study assistant, touch screen questionnaire modules, as well as biomaterial collection (34). While all participants completed a standard Level-1 assessment (L1; ∼3–4 h), a subset of approximately ∼20% of the participants was randomly selected for a more in-depth Level-2 assessment (L2; ∼5 h). Additionally, L2 assessment was administered to the subset of participants undergoing MRI assessment, resulting in a total of ∼28% of subjects undergoing L2 assessment (35). The current analyses used the baseline assessment of the first 101,667 participants (NAKO data freeze 100,000; DF 100 K) and 58,703 participants (L2: *N* = 15,556) were included in the final dataset based on the completeness of the variables of interest (see below). The variables used for the present analyses were assessed in the L1 module for all participants, besides the full NAKO MINI Classification (see below) which was only assessed in the L2 interview.

### 2.2. Measures

#### 2.2.1. Childhood trauma screener (CTS)

The Childhood Trauma Screener (CTS) (40) is a short version of the Childhood Trauma Questionnaire (CTQ) (41) measuring traumatic experiences during childhood and adolescence. In the touchscreen module, five subscales of childhood maltreatment were assessed with one item each, rated on a five-point Likert scale: *physical abuse*, *emotional abuse*, *sexual abuse*, *physical neglect*, and *emotional neglect* (CTS items). For the present analyses, items were dichotomized into no/low trauma or trauma following established procedures (37, 42). The items and thresholds for dichotomization are displayed in Table 1. Based on this categorization, additional binary variables indicating the presence of any *childhood maltreatment* (based on all CTS items), the subcategories any *abuse* (based on all abuse items), and any *neglect* (based on all neglect items) were assigned. In the main analyses, both CTS items and CTS subcategories were investigated. For sensitivity analyses, the CTS sum score was calculated (range 5–25) with higher scores indicating more severe maltreatment. Subjects with a missing value for any CTS item were excluded from analysis. For a more detailed description of the assessment and scoring of the CTS in the NAKO, see Klinger-König et al. (2022) (37).

**Table 1:** Frequency distribution of response categories to CTS items (*N* = 58,703).

	Mean	SD	Never	Rarely	Sometimes	Often	Very Often	Maltreated
Physical Abuse: People in my family hit me so hard that it left me with bruises or marks.	1.3	0.7	82.6%	10.5%	4.8%	1.3%	0.7%	6.9%
Emotional Abuse: I felt that someone in my family hated me	1.2	0.7	86.8%	6.8%	3.7%	1.6%	1.1%	6.4%
Sexual Abuse: Someone molested me (sexually).	1.1	0.4	94.8%	2.8%	1.8%	0.3%	0.3%	5.2%
Physical Neglect: There was someone to take me to the doctor if I needed it. (R)	4.2	1.2	5.3%	4.6%	12.7%	18.3%	59.1%	9.9%
Emotional Neglect: I felt loved. (R)	4.3	0.9	1.4%	5%	7.9%	34.1%	51.6%	6.3%

Responses defined as “trauma” according to Glaesmer et al. (2013) are marked in grey. (R)=Reversed coding.

#### 2.2.2. Family history of depression

In the NAKO, family history of depression was assessed as the parental history of depression: In the touchscreen module, participants were asked about a history of depression in their biological mother and father. If applicable, the age of the affected parent at the time of the diagnosis was recorded in four categories (under 40, 40–59, 60 or older, age unknown). From these answers, the number of affected parents and binary-coded family history (family history negative = no parents affected; family history positive = one or both parents affected) were assigned only for the subjects with complete information, i.e., if both biological parents were known to them, and the participants could answer for both with yes or no on the history of depression (*n* = 68,151), and subjects with information for only one parent (*n* = 13,951), or none of the parents (*n* = 19,565) were excluded. For the subjects with complete information, the earliest parental onset was assigned. In the case of participants with depression in both parents, the earlier parental onset of the two was assigned. Unknown earliest parental onset was assigned if the age of onset was unknown for at least one of the parents.

#### 2.2.3. Self-reported physician's diagnosis of depression

In the face-to-face interview, participants were asked whether they had ever been diagnosed with a depressive disorder during their lifetime or not. If applicable, the age or calendar year of the first diagnosis was assessed. Additionally, treatment of depression by a physician or psychologist during the past 12 months was recorded (yes/no).

#### 2.2.4. MINI depression classification

For lifetime depression, an adapted version of the Episode of Major Depression module of the Mini-International Neuropsychiatric Interview (MINI, German version 5.0.0) (43) was used. In the face-to-face interview, all participants (L1 and L2) answered an initial filter question about the lifetime occurrence of depression. If they answered affirmatively, the two MINI screening questions on the two cardinal symptoms of depressive disorders (depressed mood; loss of interest/pleasure in most activities) followed. If the MINI Screen was positive, only L2 participants were asked to answer the full depression module for a NAKO MINI Classification, assessing symptomatology (Criterion A) and impairment (Criterion C). In the present analyses, results are reported for the NAKO MINI Classification (L2), indicating the presence or absence of lifetime depression.

#### 2.2.5. Patient health questionnaire (PHQ-9)

Depressive symptoms during the last two weeks were measured via the touchscreen with the depression module of the Patient Health Questionnaire (PHQ-9), a validated self-report screening instrument assessing the DSM-IV symptoms of a depressive episode in nine items (44). Symptom severity is measured with the PHQ-9 sum score across all items, ranging from 0 to 27 (44). Additionally, following previous analyses in the NAKO (36), we used the commonly applied cut-off score of PHQ-9 ≥ 10 (45) to indicate the presence of a moderate depressive episode.

A more detailed description of the assessment and scoring of depression measures in the NAKO can be found in Streit et al. 2022 (36).

#### 2.2.6. Education level

Education was classified according to the International Standard Classification of Education 97 (ISCED97) (46) as reported previously for the NAKO (47). The education level of participants was categorized into lower (level 1/2), intermediate (level 3/4), and higher education (level 5/6). At the time of the analyses, the education classification was not finalized for all participants. Subsequently, all participants without a classification were excluded from the present analyses.

### 2.3. Statistical analysis

**Figure 1:**
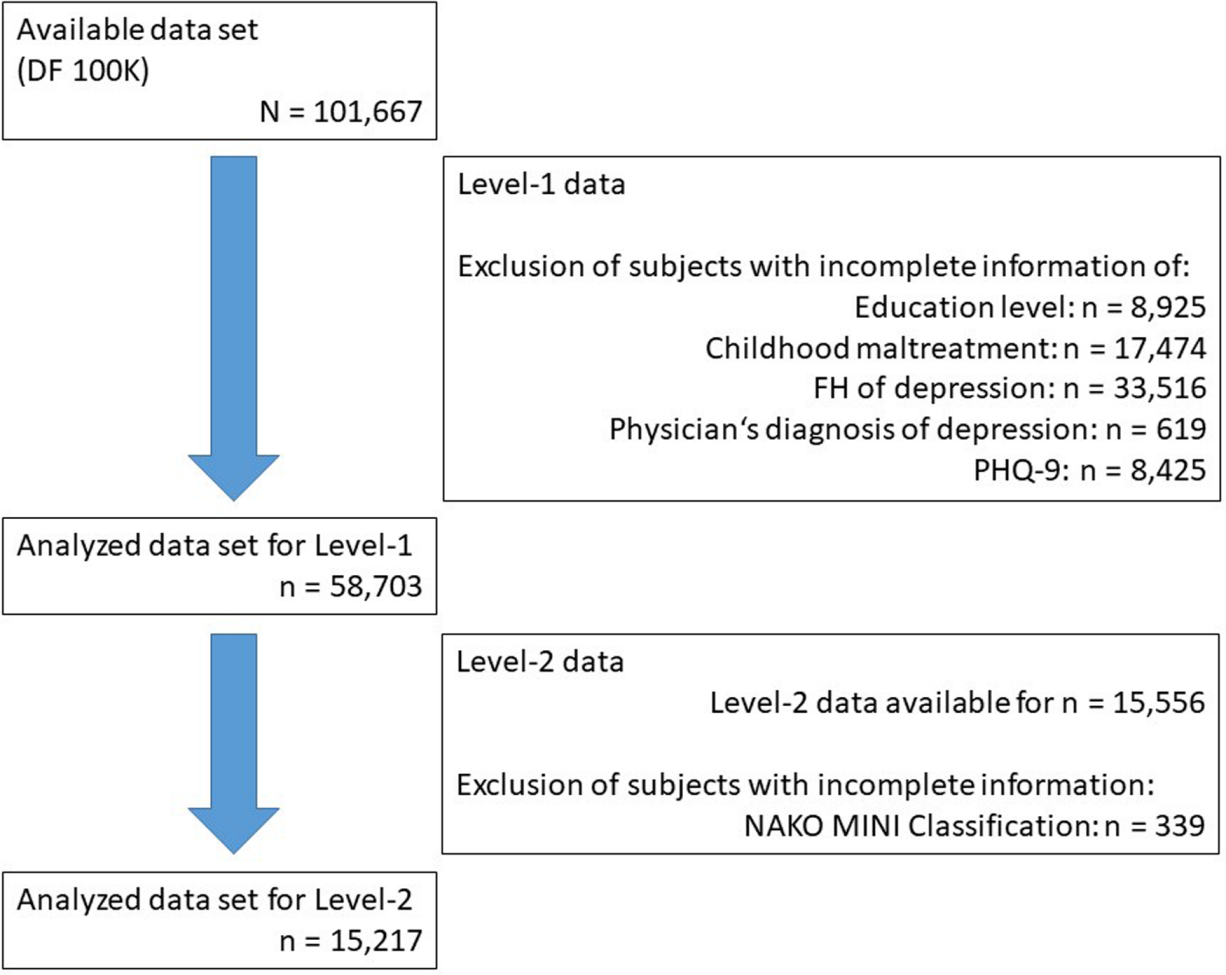
Flow chart of sample selection. The numbers of subjects excluded due to missing information, and the resulting sample sizes are indicated in the figure. Subjects with any missings on the variables of interest from the Level-1 assessment were excluded from all analyses and some subjects had multiple missings. The final sample included 58,703 subjects. Of these, 15,556 underwent the more detailed Level-2 assessment, and 15,217 had data available for the outcome “NAKO MINI Classification”.

Analyses were carried out using R (v3.5.1). Only participants with complete information on the family history of depression (*n* = 68,151), CTS (*n* = 84,193), physician's diagnosis (*n* = 101,048), PHQ-9 (*n* = 93,242), and education level (*n* = 92,742) were included in the final models (*N* = 58,703). The NAKO MINI Classification (L2) was available for *n* = 15,217 subjects in the final dataset (see Figure 1). Frequencies, mean scores, and standard deviations are reported. Associations with categorical and continuous depression outcomes were tested using logistic (logit link function) and linear regression models, respectively. Potential confounders (i.e., sex, age, age^2^, education level, study center) were included as covariates in all models. Detailed regression results and the corresponding model terms can be found in the respective Supplementary Tables (Tables S1–S68). Estimated means and frequencies, and their differences were extracted with the R package *emmeans* (48). Estimates of Odds Ratios (OR), means, and frequencies are reported with 95% confidence intervals (CI). Independent variables of interest were assessed for multicollinearity by testing the variance inflation factors of their direct effects for each regression model using the *vif* function in the R package *car* (49) (all VIF < 5). Uncorrected *p*-values are reported.

#### 2.3.1. Association of family history of depression with childhood maltreatment

To assess the association of family history of depression with childhood maltreatment, logistic models were calculated with dichotomous CTS items and CTS categories (no/low trauma vs. trauma) as the dependent, and family history of depression as the independent variable with separate models for (a) the number of affected parents (0, 1, 2) and (b) the earliest parental age at onset (no family history of depression, under 40, 40–59, 60 or older, age unknown) as a categorical independent variable. For both models, subjects reporting no family history of depression were used as the reference category.

#### 2.3.2. Association of family history of depression and childhood maltreatment with depression and potential interactions

First, separate models with the depression measures as a dependent (lifetime physician's diagnosis, the NAKO MINI Classification, the PHQ-9 sum score, and PHQ-9 ≥ 10) and the childhood maltreatment or family history of depression as independent variables were calculated, respectively. To test the joint effects between childhood maltreatment and family history of depression on depression beyond their individual effects, models with the depression measures as the dependent variable, and family history of depression (number of affected parents) and the childhood maltreatment categories *childhood maltreatment*, *abuse*, *neglect* as independent variables and the respective interaction terms were calculated.

#### 2.3.3. Mediation of family history effects on depression by childhood maltreatment

Causal mediation analyses were carried out with the R package *mediation* (50) using nonparametric bootstrapping with 1,000 simulations, testing whether childhood maltreatment mediates the associations of family history of depression on depression measures. Models were calculated with the lifetime physician's diagnosis, the NAKO MINI Classification, the PHQ-9 sum score, and PHQ-9 ≥ 10 as dependent variables, CTS items and CTS categories as binary mediators, and binary coded family history of depression (family history negative/family history positive) as the independent variable. The Total Effect, the Average Causal Mediated Effect (ACME), the Average Direct Effect (ADE), and the Mediated Proportion, i.e., the extent to which the pathway through the mediating variable accounts for the total effect, were reported (50). Additionally, stratified mediation analyses of the age groups of earliest parental depression onset (comparing the respective parental age at onset group to the subjects with negative family history of depression) were performed with the CTS categories as mediators.

## 3. Results

### 3.1. Descriptive statistics

The final sample used in the analyses consisted of *N* = 58,703 participants (mean age = 51.2 years, 53% female), with complete data on all analyzed variables, with exception of the MINI Classification, which was only assessed in the L2 subset (*n* = 15,556; see Figure 1). The distribution and frequencies of the main variables are presented in Table 2. 81.7% of participants reported having no parents affected, 16.3% having one parent affected, and 2.0% having both parents affected. For the CTS, 24.2% reported *childhood maltreatment*, 14.1% reported *abuse,* and 14.4% *neglect*. The frequency of positive responses to specific CTS items ranged from 5.2% (*sexual abuse*) to 9.9% (*physical neglect*).

**Table 2:** Descriptive statistics of depression and childhood maltreatment measures in the complete study sample, and for women and men separately.

	Total (*n* = 58,703)	Women (*n* = 31,108)	Men (*n* = 27,595)
**Demographics**			
Age in years (range: 20–72)	51.10 (12.13)	50.57 (12.06)	51.69 (12.18)
**ISCED97 Education level**			
Lower (level 1/2)	1.5%	1.9%	1.1%
Middle (level 3/4)	39.6%	44.4%	34.1%
Higher (level 5/6)	58.9%	53.7%	64.8%
**CTS items**			
Physical abuse	6.9%	6.6%	7.3%
Emotional abuse	6.4%	8.1%	4.5%
Sexual abuse	5.2%	8.0%	2.1%
Physical neglect	9.9%	9.6%	10.3%
Emotional neglect	6.3%	7.2%	5.4%
**CTS categories**			
Any maltreatment	24.2%	26.2%	22%
Any abuse	14.1%	16.9%	11.1%
Any neglect	14.4%	14.6%	14.1%
Sum Score (range: 5–25)	7.08 (2.47)	7.11 (2.66)	7.06 (2.25)
**Family history of depression**			
**Number of affected parents**			
No parents affected	81.7%	79.2%	84.6%
One parent affected	16.3%	18.5%	13.9%
Both parents affected	2.0%	2.4%	1.5%
**Parental age at onset** ^a^			
No family history	81.7%	79.2%	84.6%
Under 40	3.2%	3.9%	2.3%
40–59	6.1%	6.9%	5.2%
60 or older	5.9%	6.3%	5.4%
Unknown	3.1%	3.7%	2.5%
**Self reported physician's diagnosis**			
Lifetime diagnosis Depression	12.5%	16.1%	8.5%
Age at first diagnosis^b^ (*n* = 7,288)	40.77 (12.26)	40.17 (12.15)	42.05 (12.40)
	[10–70]	[10–69]	[10–70]
Treatment last 12 months (*n* = 58,694)	5.7%	7.6%	3.6%
**MINI Interview (L1 & L2)**			
Screen positive L1 & L2	24.4%	28.7%	19.6%
**MINI Interview (L2 only)**			
Screen positive L2 (*n* = 15,556)	23.4%	28.1%	18.8%
MINI Classification positive (*n* = 15,217)	13.8%	17.1%	10.5%
Age at onset (*n* = 2,103)^c^	36.14 (13.13)	35.9 (13.23)	36.53 (12.97)
	[11–72]	[11–72]	[11–65]
**PHQ-9**			
Sum Score (range: 0–27)	3.55 (3.46)	3.99 (3.60)	3.05 (3.22)
≥10	6.0%	7.4%	4.5%

Mean and standard deviations are given for continuous variables, and the frequency of categorical variables is given in percent. For continuous variables where the range differed between males and females, the respective ranges are reported in square brackets. CTS: childhood trauma screener. N, total number of participants in the sample; n, number of participants with valid values on the respective measures, if deviating from *N* = 58,703. L1 = Level-1 assessment completed by all subjects. L2 = variable reported for subsample that completed the more detailed Level-2 assessment (*n* = 15,556).

^a^ If both parents were affected, participants were assigned to the earlier age at onset group.

^b^ Data presented for participants with a lifetime physician's diagnosis of depression.

^c^ Data presented for the L2 participants with a positive NAKO MINI Classification. PHQ-9 = Depression Scale of the Patient Health Questionnaire (PHQ-9). Based on (NAKO data freeze 100,000; application NAKO-399).

### 3.2. Association of family history of depression with childhood maltreatment

**Figure 2:**
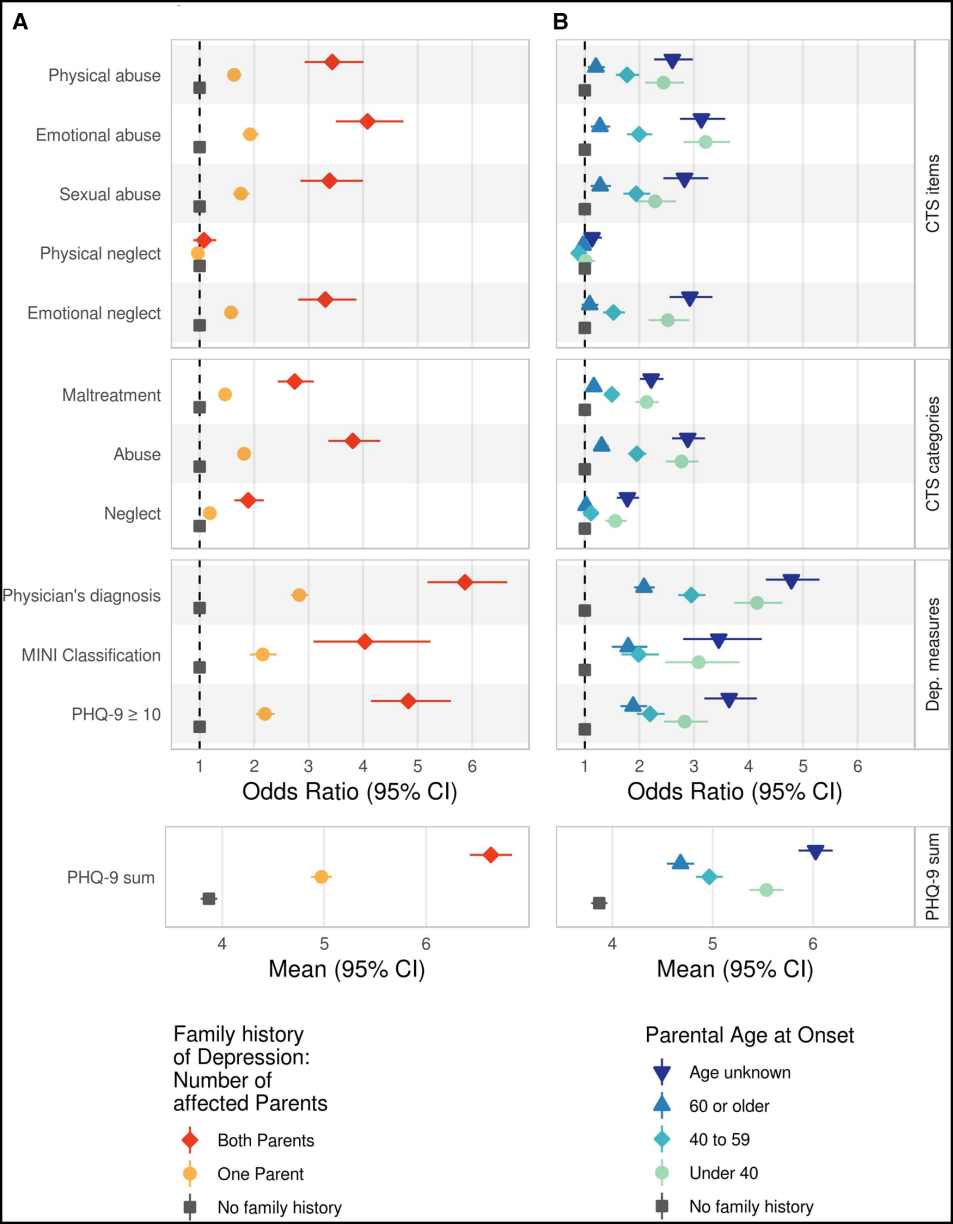
Association of measures of family history of depression with measures of childhood trauma and depression. Family history of depression is assessed as (**A**) the number of affected parents and (**B**) as the earliest onset of parental depression. Associations are shown for single CTS items, CTS categories and depression measures Odds Ratios with 95% confidence intervals are shown for categorical measures, and estimated means with 95% confidence intervals for continuous measures. As the reference group, participants reporting no parental history of depression were selected (grey square). Estimates have been adjusted for age, age^2^, sex, education level (lower/intermediate/higher), and study center. CTS items were dichotomized, and CTS subcategories were analyzed following standard procedure (37, 41). CTS, childhood trauma screener; Dep. measures, Depression measures; PHQ-9, Depression Scale of the Patient Health Questionnaire; CI, confidence interval.

The association of family history of depression with childhood maltreatment was tested with family history measures as independent and childhood maltreatment measures as dependent variables. Higher Odds Ratios of single CTS items were observed in participants with one parent affected (OR range across items = 1.58–1.93) and both parents affected (OR range across items = 2.93–4.08) compared to those with a negative family history of depression, except for the item *physical neglect* (OR range = 0.97–1.08; see Figure 2A upper panels, Supplementary Tables S1–S8). For the subcategories, a descriptively stronger association was shown for *abuse* (OR one parent affected = 1.82; OR both parents affected = 3.81) than *neglect* (OR one parent affected = 1.19; OR both parents affected = 1.89).

Furthermore, younger (<40 years; OR range = 2.29–3.22) or unknown (OR range = 2.61–3.14) age at onset of parental depression were associated with higher Odds Ratios of CTS items than older age at onset (≥ 40 years) or negative family history of depression, except for *physical neglect* (OR range = 1.01–1.13; see Figure 2B and Supplementary Tables S9–S16).

### 3.3. Associations of family history of depression and childhood maltreatment with depression and potential interactions

The association of family history of depression with depression was tested with family history measures as independent and depression measures as dependent variables. There were substantial main associations of family history of depression with all tested depression measures. Subjects with a positive family history of depression showed higher frequencies and mean scores of depression measures (all *p* < 7.1 × 10^−25^; see Figure 2A). Compared to the subjects reporting a negative family history of depression, ORs ranged from 2.18 (NAKO MINI Classification) to 2.83 (physician's diagnosis) for one parent affected, and from 4.03 (NAKO MINI Classification) to 5.87 (physician's diagnosis) for both parents affected (see Figure 2A and Supplementary Tables S17–S19). Mean scores on PHQ-9 sum scores were 3.25, 4.36, and 6.02 for negative family history of depression, one parent affected, and both parents affected, respectively (see Figure 2A and Supplementary Table S20). An association of age at onset of parental depression with all depression measures was observed with the strongest associations for age at onset under 40 (OR range = 2.83–4.16) and unknown (3.46–4.79) age at onset (Figure 2B and Supplementary Tables S21–S24).

**Figure 3:**
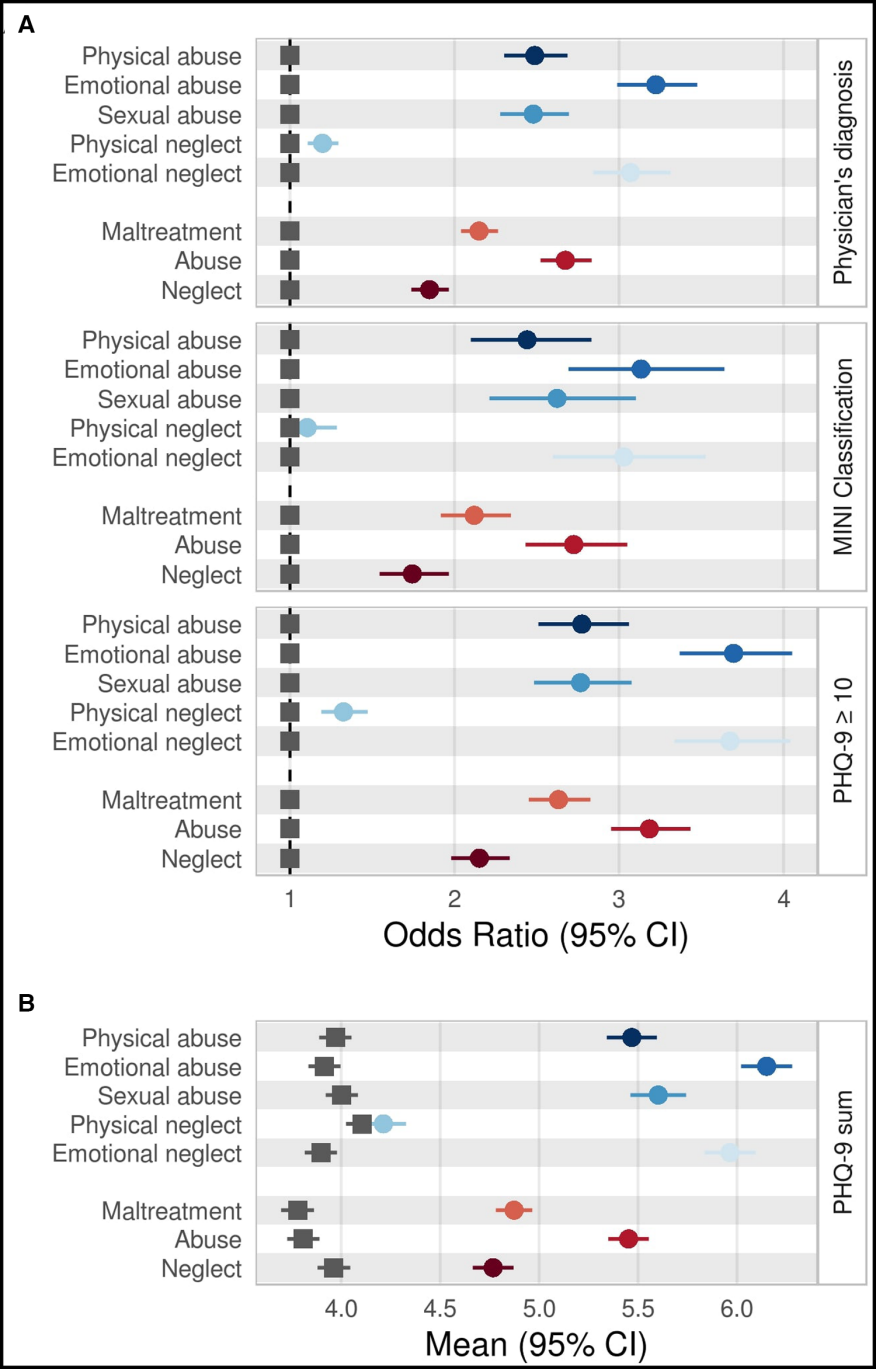
Associations of measures of childhood trauma with measures of depression. (A) Odds Ratios and (B) means with 95% confidence intervals for associations of single CTS items as well as CTS categories (childhood maltreatment, abuse, and neglect) and depression measures. As the reference group, participants who reported no/low trauma were selected (grey square). Estimates have been adjusted for age, age^2^, sex, education level (lower/intermediate/higher), and study center. CTS items and CTS categories were analyzed and dichotomized following standard procedure (37, 41). CI, confidence interval; CTS, childhood trauma screener; PHQ-9,Depression Scale of the Patient Health Questionnaire.

The association of childhood maltreatment with depression was tested with childhood maltreatment measures as independent and depression measures as dependent variables. CTS items and CTS categories were positively associated with depression measures (Figures 3A,B). Compared to low/no trauma, the OR for *childhood maltreatment* ranged from 2.12 (NAKO MINI Classification) to 2.63 (PHQ-9 ≥ 10). On a category level, a stronger association was observed for *abuse* (OR range = 2.73–3.18) than for *neglect* (OR range = 1.74–2.15). On a single-item level, the smallest associations were observed for *physical neglect* (all OR < 1.33), compared to all other CTS items (OR range = 2.44–3.70; see Figure 3 and Supplementary Tables S25–S56). For the NAKO MINI Classification, the association with *physical neglect* was non-significant (OR =  1.11).

**Figure 4:**
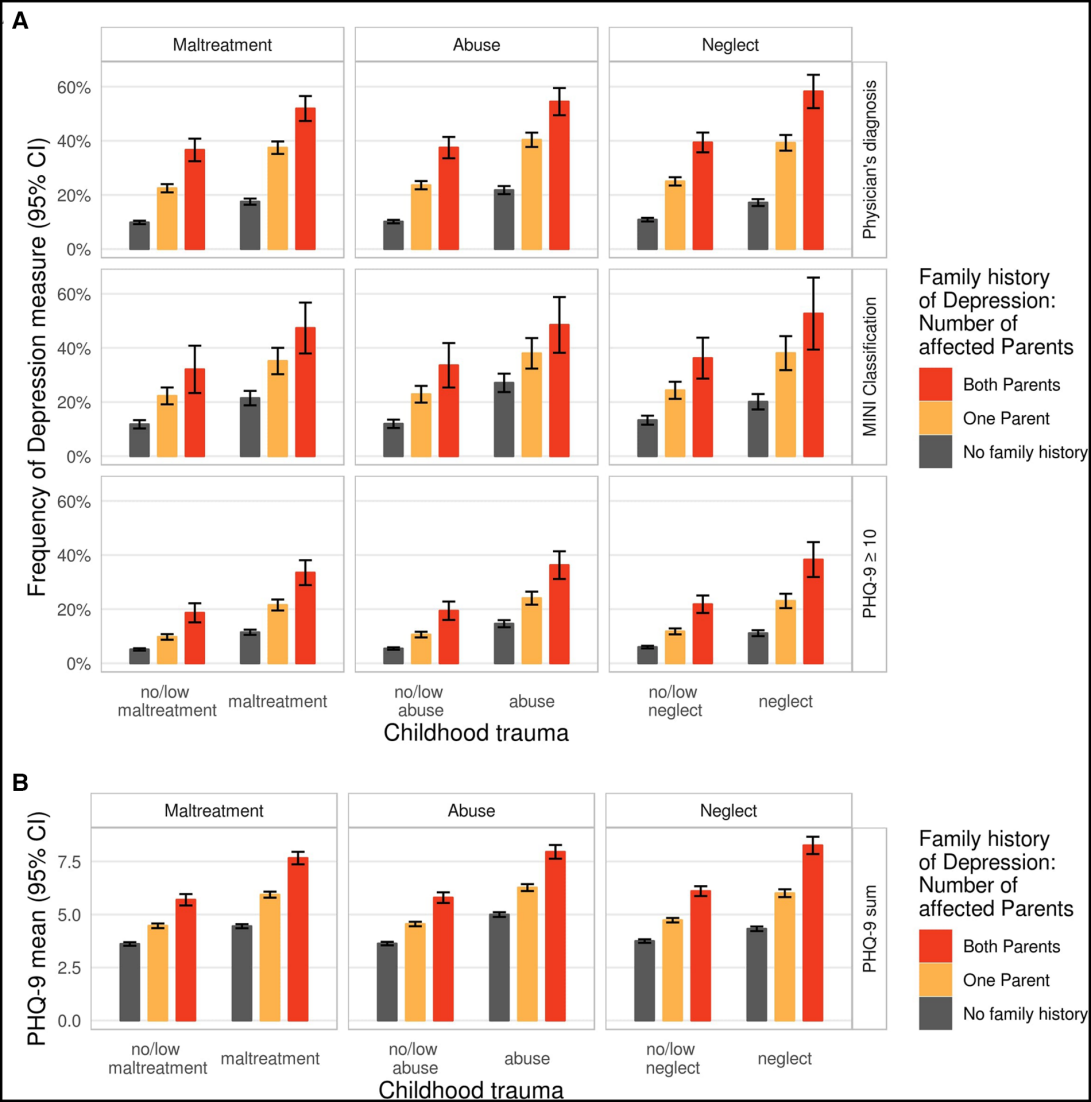
Associations and interactions of measures of childhood trauma and family history of depression with measures of depression. (A) Estimated frequencies of physician's diagnosis of depression, MINI Classification, and PHQ-9 ≥ 10, and (B) estimated mean PHQ-9 sum score by childhood maltreatment (*maltreatment*/*abuse*/*neglect*) and by family history of depression measured by the number of affected parents. Estimates have been adjusted for age, age^2^, sex, education level (lower/intermediate/higher), and study center. CI, confidence interval.

To test joint effects between childhood maltreatment and family history of depression on depression beyond their individual effects, models with family history and childhood maltreatment measures as independent variables including interaction terms and depression measures as dependent variables were tested. No significant interaction was observed for the models with binary depression outcomes (all *p* > 0.06; Supplementary Tables S57–S65). For the CTS categories, the estimated frequency of the physician's diagnosis and NAKO MINI Classification ranged from approximately 10% in subjects with negative family history of depression and childhood maltreatment to more than 45% in subjects with both parents affected and childhood maltreatment, and approximately 5% and more than 30% for PHQ-9 ≥ 10, respectively (Figure 4A). A significant interaction between family history of depression and the respective childhood maltreatment measures was observed only in the models with PHQ-9 sum scores, with stronger increases of PHQ-9 sum scores in subjects reporting both family history of depression and childhood maltreatment (Figure 4B and Supplementary Table S66–S68) (all *p* < 3.01 × 10^−06^). Here, a significant interaction for the PHQ-9 sum score was found for all CTS categories (*childhood maltreatment*, *abuse*, *neglect*). The estimated PHQ-9 sum score increase associated with *childhood maltreatment* was 0.84 [CI = 0.77–0.91] in the subject with negative family history of depression, 1.47 [1.32–1.61] in the subjects with one parent affected, and 1.97 [1.58–2.35] in the subjects with both parents affected (1.37 [1.28–1.46], 1.72 [1.55–2.56]; 2.16 [1.76–2.56] for *abuse* and 0.58 [0.49–0.67]; 1.27 [1.09–1.46]; 2.16 [1.70–2.62] for *neglect*, respectively).

### 3.4. Mediation of the association of family history of depression with depression by childhood maltreatment

**Figure 5:**
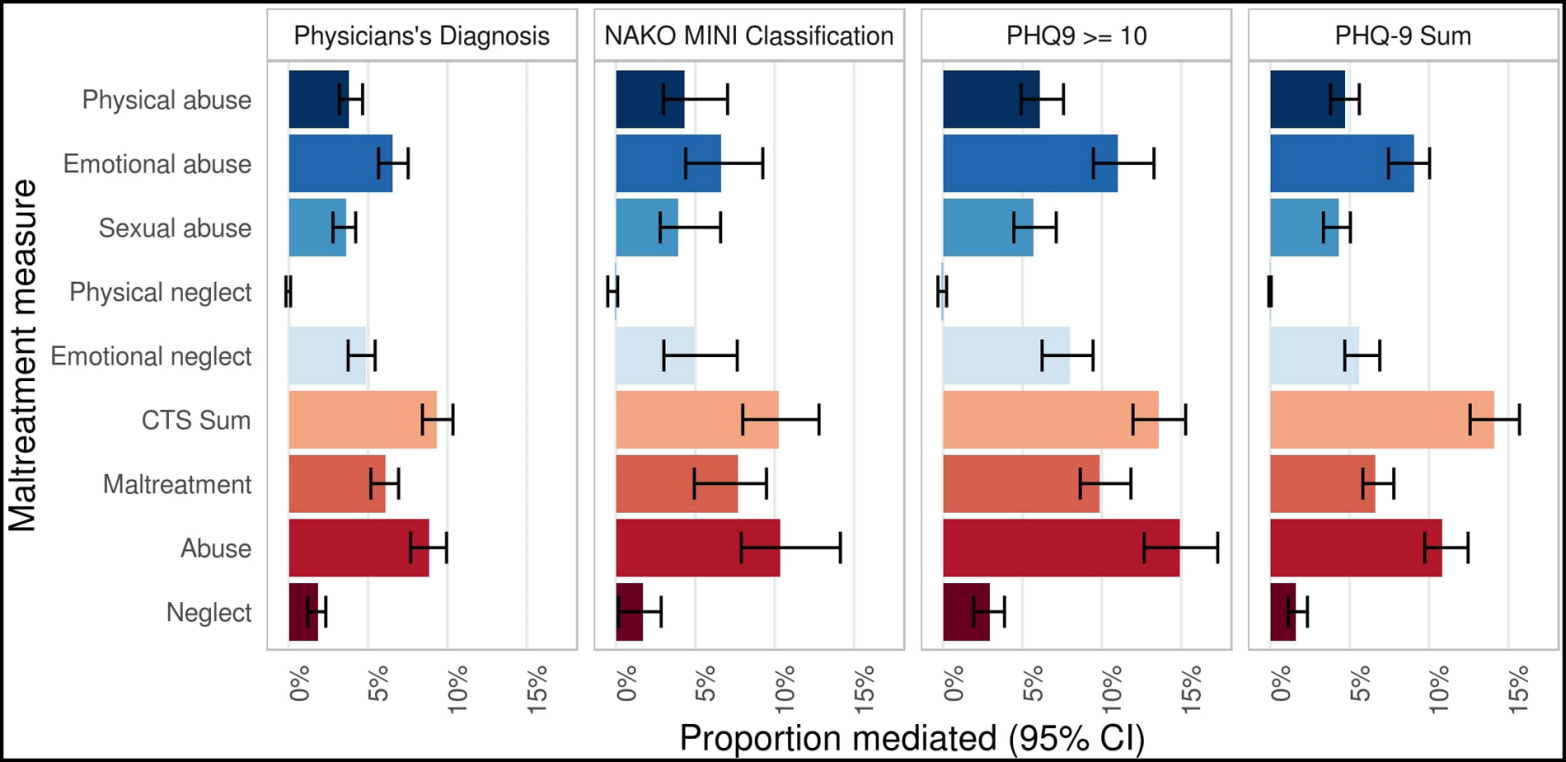
Mediation of the associations of family history of depression with depression by childhood trauma. Mediation of the associations of family history of depression with physician’ diagnosis, NAKO MINI Classification, PHQ-9 ≥ 10, and PHQ-9 sum by CTS items and CTS categories. Proportion mediated is given in percent. Analyses have been adjusted for age, age^2^, sex, education level (lower/intermediate/higher), and study center. CI, confidence interval; CTS, Childhood Trauma Screener.

To test whether childhood maltreatment mediates the associations of family history of depression with depression measures, mediation models were calculated with family history of depression as independent variables, childhood maltreatment variables as mediators, and depression measures as dependent variables. In all mediation models, we observed a significant mediation of the association of family history of depression with depression by all CTS items and CTS categories (all *p* < 0.02), except for *physical neglect* (all *p* > 0.40). The observed proportion mediated by *childhood maltreatment* was relatively small, ranging from 6.1% (physician's diagnosis; CI = 5.2%–6.9%) to 9.9% (PHQ-9 ≥ 10, CI = 8.6%–11.8%). Sensitivity analyses with the CTS summary score showed a slightly larger proportion mediated for all depression measures, ranging from 9.3% (physician's diagnosis, CI = 8.4%–10.3%) to 14.1% (PHQ-9 sum score, CI = 12.6%–15.7%). The proportion mediated was higher for *abuse* (8.8%–14.9%; range CI = 7.7%–17.3%) than for *neglect* (1.6%–2.9%; range CI = 0.1%–2.8%) on all depression measures, for details see Figure 5 and Supplementary Tables S69–S104. Stratified analyses with parental age at onset indicated that the proportion mediated was descriptively highest in the subjects whose parents had a depression onset of under 40 years (*childhood maltreatment *= 7.8%–14.8%, range CI = 5.3%–17.6%, all *p* < 0.001) or an unknown age at onset (*childhood maltreatment *= 6.9%–12.6%, range CI = 5.6%–15%, all *p* < 0.001). The lowest proportion mediated was observed in the subjects whose parents had a depression onset ≥60 years (*childhood maltreatment *= 2.9%–4.4%; range CI = 0.1%–8.4%, all *p* < 0.026 see Supplementary Figure S1 and Tables S105–S152).

## 4. Discussion

We investigated the interplay between family history of depression and childhood maltreatment in relation to depression in a large, population-based cohort. The present study confirms the strong associations of family history of depression and childhood maltreatment with depression (6, 51–53) with both factors found to be more prevalent in participants with depression. The interaction analyses provide little evidence that either factor modulates how strongly the other factor influences the risk of developing depression, with only interactions being found for the continuous measurement of current depressive symptoms (PHQ-9 sum score). The results of the mediation analyses are compatible with a mechanistic model which assumes that the effects of family history of depression on the later development of depression may be in part mediated by childhood maltreatment. However, interpretation needs to be cautioned as the data was assessed in a cross-sectional design, with data being evaluated retrospectively. Younger or unknown parental age at onset was associated with higher frequencies of childhood maltreatment, higher frequencies and mean values of depression outcomes, and a stronger mediation of the association of family history of depression with depression measures by childhood maltreatment.

The higher levels of childhood maltreatment in participants who reported a positive family history of depression are in line with previous findings (17, 54). A broad range of risk factors linked both to a positive family history of depression and childhood maltreatment might drive this association. A positive family history of depression might affect the parent-child interaction, e.g., by a more hostile or disengaged parenting style (55, 56), but might also be more broadly linked to other risks for childhood maltreatment such as low education level or racial discrimination (15). Furthermore, childhood trauma itself has been shown to have a genetic component in the sense of gene-environment correlations (57, 58). This component is genetically correlated with depression risk (58), which might contribute to the observed association between family history of depression and childhood maltreatment. The increased childhood maltreatment in subjects with both parents affected might therefore be associated with a higher genetic burden, but also by a stronger impact of direct parental effects, with the lack of compensatory influence of a second healthy parent, or an increased burden of other risk factors related to parental depression and childhood maltreatment (56). The higher levels of childhood maltreatment in participants with a lower parental age at onset (≤40) might reflect that they were more likely to have experienced their parents' depression during childhood and adolescence, the time frame assessed in the CTS (40). Previous research indicates that a more fine grained analysis of the timing of childhood maltreatment, might further inform on the associations. For example, a study by Dunn et al. points towards stronger effects of early childhood trauma (age 0–5) on later-life depression compared to childhood trauma in later developmental phases (59). Other studies suggest that the impact of childhood trauma on mental health depends on the neurodevelopmental stage of the child at the time of exposure; potentially affecting the development of brain structures that are involved in affective disorders (60, 61). Information regarding the timing of childhood trauma would have allowed for more refined analyses in the present sample.

Regarding the similarly strong relationships between family history of depression and both childhood maltreatment and depression in participants who indicated an unknown parental age at onset, it can be speculated that the parental onset was often before birth or at a young age of the participants. Hence, the parental background might be similar to those with an age at onset under 40. Alternatively, an unknown age at onset could also reflect less communication about parental depression in the family.

In addition, the present analyses extend the findings of previous studies on the association of family history of depression (36), and childhood maltreatment (37) with depression in the NAKO. Associations of family history of depression were stronger in participants with both parents affected, which is consistent with a prior meta-analysis reporting a higher risk for depression in subjects with two affected first-degree relatives compared to subjects with one affected first-degree relative (9), with the ORs in the present study being even more pronounced than the associations in the meta-analysis. Additionally, earlier or unknown age at onset of parental depression was associated with an increased risk of depression. As discussed above, this can be interpreted in the sense of an increased (genetic) risk burden (62), but might also be linked to timing effects (59, 63), and other social determinants (64, 65).

Individuals who experienced childhood trauma were more likely to report lifetime depression or depressive symptoms, with stronger associations observed for *abuse* than for *neglect.* In agreement with the present item-specific findings, a prior meta-analysis on the association between CTQ scores and depression showed the strongest associations with *emotional abuse* and *emotional neglect* (25). It has been hypothesized that emotional maltreatment is more strongly related to internalizing symptoms (e.g., sadness, fatigue) common in depression than other types of maltreatment, potentially accounting for the strong association with depression outcomes (66).

Family history of depression and childhood trauma might influence depression risk by shaping long-term functioning of a broad range of neurobiological systems. For example, early traumatic experiences have been shown to program the functioning of the hypothalamic–pituitary–adrenal (HPA) axis, a central regulator of the stress response, impacting responsiveness to stress in later life (60, 61, 67, 68). In line with that, parental depression has been linked to the alterations in the offsprings' regulation of the HPA axis (69). Additionally, childhood maltreatment has been linked to increased systemic inflammation and inflammatory biomarkers (70–73). In turn, increased inflammation has been associated with an elevated risk of depression (70–72). Furthermore, early maltreatment and a positive family history of depression both might affect the neurodevelopment. Both risk factors have been linked to alterations in the structure or function of brain regions such as the amygdala and the hippocampus (60, 61, 67, 68), which are important for the regulation of stress and emotions (74), and show associations with depression (68, 75). The described mechanisms are not mutually exclusive and may influence the effects of family history of depression and childhood maltreatment on depression conjointly.

It has been suggested that subjects with a predisposition for depression, such as a positive family history of depression, might be at a particularly high risk of developing depression if they have been exposed to childhood trauma. In the present study, no evidence for a statistical interaction of family history of depression and childhood maltreatment on measures of lifetime depression was found. For current depressive symptoms, and limited to the models with the PHQ-9 sum score as the outcome, a stronger association of maltreatment was observed in the subjects reporting a positive family history of depression. Discrepancies between the different depression measures, especially in the comparison between categorical PHQ-9 ≥ 10 and continuous PHQ-9 sum score, both widely used in research (45, 76), indicate a potential influence of the exact measurements on the detectability of interactions. Moreover, continuous variables provide higher sensitivity and statistical power than categorical variables, allowing for better detection of associations, even if these associations have a small magnitude. Although statistically significant, the interactions in the present analyses were rather small. These results point to primarily independent associations of family history of depression and childhood maltreatment with depression, i.e., each factor increases the risk for depression largely independently of the other.

The effect of family history of depression on all depression measures was to a smaller extent mediated by the CTS categories and items, except for *physical neglect*. Larger mediation effects were observed in the subgroup of subjects reporting a younger or unknown age at the onset of parental depression. However, the proportions of the mediated effect were substantially smaller than the 35% previously reported by Jansen et al. (2016) (17). These differences might be explained by different characteristics of the investigated samples: While both studies assessed current depressive symptoms, lifetime depression was only assessed in the NAKO cohort. Additionally, Jansen et al. (2016) investigated younger subjects and included both patients with either bipolar disorder or major depression. In the NAKO cohort, bipolar disorder was not assessed but, in regard to its relatively low prevalence, was likely only present in a very small proportion of the participants (17).

As discussed above, there is a range of neurobiological pathways potentially underlying the mediation of family history effects on depression risk by childhood maltreatment. For example, parenting styles associated with a parental risk for depression (54, 55) have been linked to alterations in HPA axis (71) and immune system (77). Those systems have also been reported to be affected by childhood maltreatment (77), and alterations have been shown in individuals with a diagnosis of depression (72, 78). Similarly, it is likely that family history of depression and childhood maltreatment shape neurodevelopment, and thereby affect depression risk. Integrating neuroimaging data with the present analyses has the potential to further inform on the interplay of those factors.

Notably, the proportions mediated varied over different types of maltreatment, with the strongest mediations being observed for emotional abuse, and then emotional neglect. Correspondingly, those types were also most strongly associated with the depression outcomes, as reported previously (25). It has been proposed that specifically emotional maltreatment might shape a cognitive style with negative self-referential processing, thereby increasing depression risk (79, 80).

Overall, the present results might be affected by the measures used within this study: The depression measures capture different aspects of depression and are not interchangeable (36). Moreover, the CTS is a short 5-item screening questionnaire that does not assess all types of trauma and each of the five subtypes is only captured by a single item. Therefore, specific aspects of these subtypes might not be captured equally well with this approach, and replication of the present results in samples using the full CTS would be desirable. Additionally, the CTS does not record specific information such as the age at the time of the childhood trauma, which might be an important influencing factor for the effects of trauma on later depression (59).

Nevertheless, the CTS is an efficient tool for the economic retrospective assessment of childhood maltreatment which facilitates its widespread use in large epidemiological studies (40). At the single-item level, we observed no or only small associations of *physical neglect*. While smaller associations with mental health measures are often observed for this dimension (25), it is unclear to what degree the associations in the present study related to the limited psychometric properties of the item (40, 81), and to what degree interpretation of the *neglect* category is limited by this.

Information on the timing and severity of a positive family history of depression are also limited, as the parental age at onset was only assessed categorically in age groups, and the current age or birth year of the parents was not assessed. A more detailed assessment of family history of depression would have provided a more accurate estimate of familial genetic load (54) as well as timing of childhood maltreatment (59, 63) and would have allowed for more sophisticated familial risk assessments such as weighted family genetic risk scores (FGRS) (82). By including only subjects with available information on parental depression from both parents, and therefore inter alia excluding subjects with e.g., an unknown parent, or unwilling to report on their family history of depression, there is also a potential selection bias in the final sample. The present analyses included only participants with complete data on the investigated variables, which can result in both up and downward bias of the resulting estimates. For example, participants from single parent families or orphans may have been more likely to be excluded from the analyses. Future studies should extend the analyses to assess the characteristics of subjects with missing data, e.g., subjects choosing not to report childhood trauma, or with (partially) missing information on parental depression.

Caution is also warranted with regards to the self-report nature of the data used in the study. Self-report measures might be affected by bias such as imperfect recall (83). The participants' prevalences of lifetime depression, depressive symptoms and family history of depression in the NAKO cohort are comparable with other estimates from the German population (84–86), and their associations with sociodemographic factors are as expected (36). However, women were more likely to report a positive family history of depression than men (20.8% vs. 15.4%), which may not reflect a real difference. The higher number might be influenced by informant characteristics, such as increased sensitivity in women to mental health issues or greater knowledge about their parents' mental health history (87, 88).

Moreover, compared to a recent meta-analysis on the global average by Solmi et al. (89), a higher age at onset of depression is reported for the participants and especially their parents in the present study. For the latter, we cannot exclude a reporting bias towards an older age. However, the NAKO cohort features primarily middle-aged and older subjects with an average age of 52 years with correspondingly older parents. Reported ages at onset are more in line with results from other studies in samples with comparable age structures (90, 91). Additionally, especially parents of younger NAKO participants still might develop depression following the study assessment. As a result, some participants might have a genetic predisposition to depression that was not captured by the assessment of family history of depression in the present study.

Large population-based cohorts such as the NAKO represent a valuable resource to investigate the interplay of risk factors for mental disorders in the population (33). The broad range of depression measures provides a wealth of individual phenotypes and allows for in-depth investigations of different risk factors on various depression outcomes. The results of the mediation analyses stratified by parental age at onset underline the potential of subgroup analyses in large-scale cohorts.

Future investigations should extend the present analyses to characteristics of the symptoms or course of depression (92, 93) and investigate the associations in further subgroups, e.g., by sex or age. Additionally, potential mediators of risk such as neurological (68, 75, 94), psychological (95–97), neurocognitive (98), or immunological and endocrine functioning (70–72) should be investigated to improve the understanding of the contribution of family history of depression and childhood maltreatment to depression risk. The results of the present study suggest that the investigated subtypes of maltreatment differ in their association with family history of depression, depression outcome, and the proportion mediated by them. Therefore, future studies should address to what degree potential neurobiological mechanisms are specific to them. Moreover, while the present analyses focused on depression measures as outcomes, both a positive family history of depression and childhood maltreatment constitute fairly broad risk factors, and future research should incorporate further mental or somatic disorders (99, 100). In the future, the full data set of the NAKO study with 205.000 participants will become available and data will be linked to electronic health records of the participants, providing additional measures of mental disorders. Additionally, the present results should be replicated in other cohorts with available prospective measures of childhood maltreatment as well as in other populations to ensure robustness and generalizability of results.

The present study might guide future molecular genetic studies: It has been suggested that individual differences in the impact of childhood maltreatment can be explained by gene-environment interactions (G x E) in which the genetic makeup influences the impact of environmental factors on disease risk (101). The present study assessed family history of depression as a marker for genetic risk (11, 12) which also encompasses environmental factors shared within a family (102). As a more direct measure of genetic risk, recent studies have used genome-wide data to model GxE interactions. Polygenic scores (PGS) integrating the genetic risk associated with single variants (SNPs) into one weighted composite score (103, 104) have been shown to be linked to a positive family history of mental disorders in healthy subjects, as well as to a positive family history of mental disorders in patients with mental disorders (105–107) while showing a partially independent contribution to disorder risk (108–111). Molecular genetic investigations integrating genotypic and family data (112, 113) will help to dissect the genetic and non-genetic pathways through which family history of depression and childhood maltreatment contribute to depression risk. However, the present analyses indicate that interactions between those factors might be small, will therefore require very large samples (114), and also might depend on the investigated measures. Genetic data becoming available in the NAKO will contribute to such investigations.

The present study is of potential interest to health policymakers, as it provides further evidence that childhood maltreatment is a major risk factor for depression (19, 52, 53). Li and colleagues (18) suggested that a reduction of childhood maltreatment at the population level may prevent a large proportion of depression cases worldwide, stressing the importance of more effective child protection policies. Additionally, we found an increased likelihood of childhood maltreatment in individuals with a positive family history of depression. While a genetic risk for depression is not modifiable, awareness of these risks for early intervention, communication about and coping with parental mental disorders within the family should be improved with support programs for affected families. Children of affected parents could be targeted specifically with protective measures and monitored more closely for early signs of childhood maltreatment. While largely not interacting in a statistical sense, the independent associations of family history of depression and childhood maltreatment make individuals with both risk factors especially vulnerable to developing depression. Further understanding of the factors determining the risk of those individuals as well as of potentially protective factors would help to improve prevention and treatment.

## Data Availability

Publicly available datasets were analyzed in this study. This data can be found here: https://transfer.nako.de/transfer/index.
