# Supplementary material for: The interplay of family history of depression and early trauma: associations with lifetime and current depression in the German national cohort (NAKO)
Source: Front Epidemiol. 2023 May 23;3:1099235. doi: 10.3389/fepid.2023.1099235 (PMC10959537; doi:10.3389/fepid.2023.1099235)
Supplement: Supplementary file 2 [file Image1.pdf]

Mediation of FH effects on depression by CT - stratified by age

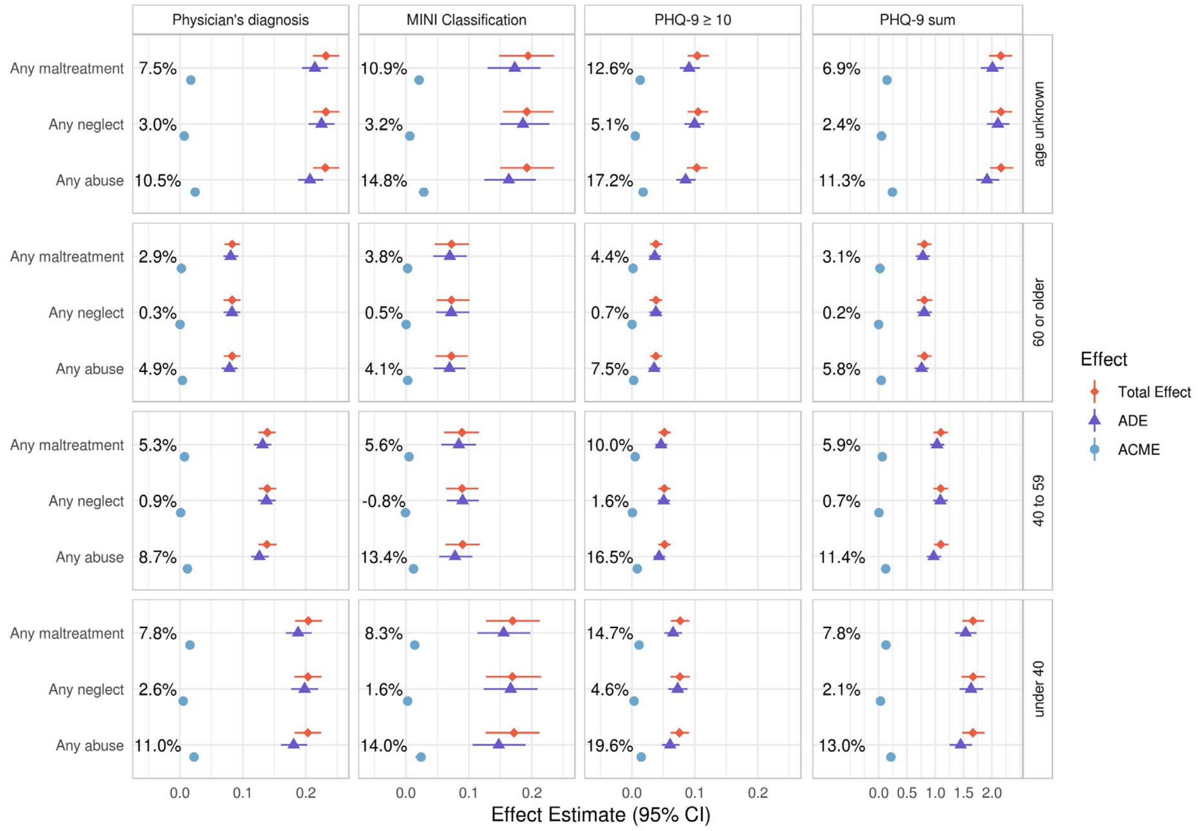

Supp. Figure S1: Mediation of FH effects by CT on Physician’s diagnosis, NAKO Mini Classification, and PHQ-9 ≥ 10 by age at onset of parental depression. Proportion mediated is given in percent. ADE = Average Direct Effect, ACME = Average Causal Mediated Effect.
